# Emerging Therapies and Advances in Sickle Cell Disease with a Focus on Renal Manifestations

**DOI:** 10.34067/KID.0000000000000162

**Published:** 2023-05-31

**Authors:** Mofiyin Obadina, Sam Wilson, Vimal K. Derebail, Jane Little

**Affiliations:** 1 Division of Hematology, Department of Medicine, University of North Carolina, Chapel Hill, North Carolina; 2 UNC Blood Research Center, University of North Carolina, Chapel Hill, North Carolina; 3 Division of Nephrology and Hypertension, Department of Medicine, University of North Carolina, Chapel Hill, North Carolina

**Keywords:** CKD, vascular

## Abstract

The underlying mechanisms of disease in sickle cell disease (SCD) contribute to a multifaceted nephropathy, commonly manifested as albuminuria. In severe SCD genotypes (*e.g.*, Hemoglobin SS [HbSS]), albuminuria and CKD are major predictors of mortality in this population. Therefore, the monitoring and management of renal function is an intrinsic part of comprehensive care in SCD. Management of nephropathy in SCD can be accomplished with SCD-directed therapies and/or CKD-directed therapies. In the past 5 years, novel disease-modifying and palliative therapies have been approved in SCD to target aspects of the disease, such as anemia, inflammation, and vasculopathy. Along with conventional hydroxyurea and chronic transfusion, l-glutamine, crizanlizumab, and voxelotor have all been shown to mitigate some adverse effect of SCD, and their effect on nephropathy is being investigated. CKD-directed therapies such as renin–angiotensin–aldosterone system blockers have long been used in SCD nephropathy; however, more complete long-term studies on benefits are needed. Given the effect of renal disease on survival, further assessment of the mechanisms and efficacy of these SCD-directed or CKD-directed therapeutic agents is essential.

## Introduction

Sickle cell disease (SCD) is a highly prevalent inherited blood disorder worldwide occurring in 300,000 births annually.^1^ It comprises sickle cell anemia (SCA) and variant SCD (compound heterozygous Hemoglobin SC or HbSb^+^ thalassemia), disorders with genotypic and phenotypic variability arising from a mutation in the hemoglobin B globin gene that produces sickle hemoglobin (HbS). Deoxygenated HbS (deoxy-HbS) polymerizes, resulting in fragile red blood cells (RBCs), chronic hemolysis with anemia and inflammation, vasculopathy, intermittent vaso-occlusive episodes (VOEs), and ultimately chronic multiorgan damage.^2^ In the more clinically severe SCA (homozygous HbSS or HbSb^0^ thalassemia), a multiorgan vasculopathy predominates and carries an increased risk of premature death.^3^ Advances in pediatric care in the United States^4–8^ has shifted the burden of disease to this new population of young adults with SCD who face a shorter lifespan compared with the general population.^9,10^ In this population, kidney disease is prevalent and can progress to CKD which affects mortality. The goal of this review is to discuss advances in SCD-focused therapies and their efficacy in reversing or slowing progression of CKD. In addition, we will describe the role of specific CKD-directed therapies in sickle nephropathy.

## Renal Manifestation in SCD

**Figure 1:**
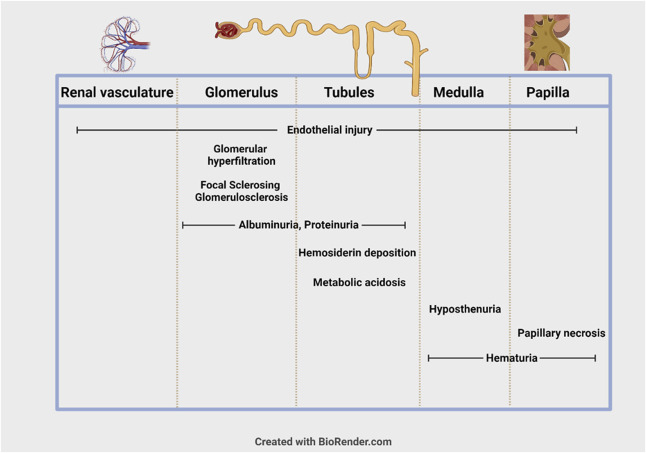
**Clinical manifestation of SCA by region of the kidneys.** SCA, sickle cell anemia.

The kidneys are sensitive to chronic hemolysis, anemia, and vaso-occlusion that are hallmarks of SCD, resulting in regional dysfunction^11–13^ in the kidney (Figure 1).

The renal cortex has a relatively low rate of oxygen extraction but receives a high rate of blood flow, which facilitates high oxygen consumption,^14,15^ making this region sensitive to hypoxia and a likely site for deoxy-HbS polymerization. The renal medullary environment is hypoxic, acidotic, and hyperosmotic, contributing to HbS polymerization and recurrent vaso-occlusion, resulting in ischemia there and in the adjacent renal papillae.^13,16^

In the glomerulus, there is evidence for endothelial injury in the arterioles and glomerular hyperfiltration, believed to be stimulated by anemia, vasodilation, and medullary ischemia.^16–19^ Free hemoglobin from intravascular hemolysis is directly toxic to the renal tubules through oxidative damage, inflammation, and depletion of nitric oxide (NO) with resultant vasculopathy.^20–22^ In addition, free hemoglobin in the proximal tubules likely reduces albumin reabsorption contributing to proteinuria.^23^ Studies show that iron deposition in the tubular epithelium^24–26^ in SCA are consistent with hemolysis, not chronic transfusion, as the proximate cause of this insult. Cellular damage to the proximal and distal convoluted tubules has been described, and there is a clinical association between hemolysis and albuminuria in this population.^27,28^

Sickle cell nephropathy can therefore appear as glomerular hyperfiltration, albuminuria, FSGS, impaired urinary concentration ability (hyposthenuria), metabolic acidosis, hematuria, papillary necrosis, and/or CKD (defined as depressed eGFR and/or proteinuria).^16,29^ Albuminuria, generally accepted as an early indicator of glomerular injury, can be seen in up to 30% of children and 60% of adults with HbSS, and associates with increasing age.^30,31^ In a longitudinal, prospective multicenter study with children and adults, albuminuria at >100 mg/g creatinine was associated with a significantly increased risk of persistent albuminuria and a faster rate of decline in eGFR.^32^ Persistence of albuminuria predicts the risk of progression of renal disease; thus, early detection of albuminuria and initiation of therapy to halt progression is the general approach to managing sickle cell nephropathy. Genetic modifiers that decrease risk of progression to CKD include higher fetal hemoglobin levels and coinherited *α*-thalassemia deletions which have net effect of decreasing the sickling effect of HbS. Genetic modifiers that increase the risk of progression to CKD include variants in *MYH9*, *APOL1*, and *HMOX1.*^33–35^ Of note, albuminuria and CKD are common in variant SCD, with or without concomitant diabetes, hypertension, or other glomerulopathy.^31^ Before determining that a patient has SCD-related kidney disease, clinicians should exclude other potential causes of non–sickle cell–related kidney disease in people with SCD.

CKD is a significant predictor of risk of death in patients with SCD^36–39^ and was present in 12% of an observational cohort (*N*=1056) of people living with SCA, with a mean age of onset of 37 years. In this cohort, most deaths (73.3%) occurred in people with prior organ damage, and renal failure was present in nearly one-third (29.4%) of those patients; CKD predicted death with an adjusted odds ratio (OR) of 2.04 (95% confidence interval [CI], 1.27 to 3.26).^40^

## SCD-Directed Therapeutics in Nephropathy

### Hydroxyurea

Hydroxyurea, the cornerstone of disease-modifying therapy in SCA, was approved by the US Food and Drug Administration (FDA) in 1998, initially for the indication of reducing frequency of painful VOEs in SCA.^41^ Hydroxyurea is a ribonucleotide reductase inhibitor that exerts its effect through multiple mechanisms, including increasing expression of fetal hemoglobin, reducing white blood cell and platelet counts, and increasing NO levels.^42^ Cumulatively, hydroxyurea decreases RBC sickling, thereby likely reducing downstream endothelial injury, intravascular hemolysis, and depletion of endogenous NO. Hydroxyurea use has been associated with reduced frequency of acute chest syndrome, reduced need for blood transfusions, likely improved mortality,^43,44^ decreased prevalence, and/or improvement of albuminuria.^45–48^ A prospective study exploring the efficacy of hydroxyurea and angiotensin-converting enzyme inhibitors (ACEi) in 191 children with SCA, after a mean follow-up of 2.19 years, showed normalization of microalbuminuria in 44% of patients on hydroxyurea. In that study, 16 of 17 hydroxyurea-treated patients without microalbuminuria at the start of the study remained albuminuria-free throughout.^49^ Another cross-sectional study of albuminuria in 149 adults with SCD found a lower prevalence of albuminuria among patients on hydroxyurea; multivariate analyses showed hydroxyurea was associated with a lower likelihood of albuminuria (OR, 0.28; 95% CI, 0.11 to 0.75; *P* = 0.01).^50^ However, smaller shorter observational studies have not always noted significant benefit.^51,52^

Studies assessing hydroxyurea's effect on eGFR have showed conflicting results.^53^ The Pediatric Hydroxyurea Phase III Clinical Trial,^54^ a multicenter, phase III placebo-controlled study of 193 children with SCA, did not identify significant differences in eGFR between the two groups. However, they did note improve urine concentrating capacity in hydroxyurea-treated children, perhaps because of preserved tubular function. These results are limited by the young cohort (mean age 13.8 months) and short follow-up period.

The timing and dose of hydroxyurea initiation may affect albuminuria outcomes. In a retrospective analysis of data from 88 children with SCA,^48^ children who started hydroxyurea before age 10 years were less likely to develop albuminuria, compared with those who started hydroxyurea after 10 years (hazard ratio, 0.49; 95% CI, 0.25 to 0.97; *P* = 0.038); children on hydroxyurea in whom baseline albuminuria resolved received a higher dose of hydroxyurea (26.7±3.8 mg/kg per day versus 21.1±3.9 mg/kg per day; *P* = 0.003). Larger controlled studies including adult patients are needed.

### Transfusion

Chronic RBC transfusions (simple and exchange) can increase hemoglobin levels and decrease the amount of sickle RBCs in circulation, which has been shown to reduce markers of endothelial injury.^55^ Older studies have demonstrated improved urinary concentration ability with simple transfusions; however, the capacity for recovery might be reduced with age.^56,57^ A retrospective review of 120 children and young adults with SCD found that patients who started chronic transfusion therapy at a younger age had a lower prevalence of microalbuminuria.^52^ They concluded that chronic transfusions were renoprotective when started younger than 9 years, while acknowledging significant transfusion-related adverse effects limiting routine use. However, another retrospective study evaluating risk of CKD progression in children did not note significant differences in the prevalence of microalbuminuria among treatment groups of hydroxyurea, chronic transfusions, or observation.^51^

### l-Glutamine

l-glutamine was approved by the FDA in 2017, with the indication of reducing frequency of VOEs.^58^ l-glutamine is an essential amino acid, and its mechanism of action in SCD is unknown. l-glutamine is essential for the synthesis of antioxidants NAD and GSH. As part of the oxidative stress that leads to hemolysis of HbS-containing RBCs, there is increased production of reactive oxidant species and free radicals that consume reduced NAD (reduced nicotinamide adenine dinucleotide) and GSH.^59–61^ The depletion of reduced nicotinamide adenine dinucleotide and GSH is mitigated by glutamine, which is believed to decrease RBC damage and the threshold for hemolysis. Although one study found significantly decreased markers of hemolysis (reticulocytes and lactate dehydrogenase) in 19 patients on l-glutamine,^62^ the phase III approval study did not reproduce this finding.^58^ Previous studies support the role of glutamine in improving NAD redox potential and decreasing endothelial adhesion.^61^ Observational studies have focused on l-glutamine's effect on pain^63^ and other complications, and its effect on progression of renal disease in SCD is unknown.

Other reviews have discussed alternative mechanisms by which glutamine could affect erythrocytes.^64^ These include the suppression of inflammatory pathways and modulation of intestinal microbiome and barrier function to create a less aggressive SCD phenotype^60^ and glutamine acting to reduce resting energy expenditure.^65^ As with all supplements, caution should be used in patients with underlying liver disease or renal disease. *Post hoc* analysis of a randomized clinical trial assessing the use of l-glutamine in critically ill patients identified the greatest potential for harm in those with evidence for renal dysfunction at enrollment into the study.^66^

### Crizanlizumab

The monoclonal antibody crizanlizumab was approved by the FDA in 2019, with the indication of reducing VOE frequency.^67^ Crizanlizumab inhibits P-selectin binding to its ligands, presumably blocking P-selectin–mediated adhesion of inflammatory cells to the endothelium, thus reducing vaso-occlusion. Given its recent approval, there are ongoing evaluations^68^ into its clinical benefits, including its effect on nephropathy. The Study Exploring the Effect of Crizanlizumab on Kidney Function in Patients With Chronic Kidney Disease Caused by Sickle Cell Disease trial (NCT04053764) is a phase II study comparing crizanlizumab and standard of care with standard of care alone in patients with SCD-associated CKD. It is based on previous studies showing increased P-selectin expression in kidney ischemia-reperfusion injury.^69^ Its primary end point is the proportion of patients who experience ≥30% decrease in albuminuria at 12 months. In addition, the Study of Two Doses of Crizanlizumab Versus Placebo in Adolescent and Adult Sickle Cell Disease Patients trial (NCT03814746) is a phase III placebo-controlled trial comparing efficacy and safety of crizanlizumab doses with a secondary outcome of albuminuria prevalence.

### Voxelotor

Voxelotor was approved by the FDA in 2019, with the indication of increasing hemoglobin concentration.^70^ Voxelotor selectively and reversibly binds to hemoglobin, keeping it longer in the oxy-Hb or R-state. This results in delayed and decreased HbS polymerization, with less hemolysis and a modest increase in Hb concentration. The results from the long-term follow-up of the initial Hemoglobin Oxygen Affinity Modulation to Inhibit HbS Polymerization study^71^ confirmed a durable increase in hemoglobin and noted significant improvement in markers of hemolysis (lactate dehydrogenase, indirect bilirubin, and reticulocytes) in the group randomized to 1500 mg of voxelotor. Given the noted effect on hemolysis markers,^72,73^ a small retrospective study evaluated albuminuria before and after voxelotor initiation in ten adult patients with SCA.^74^ They reported a 25% reduction in albuminuria from baseline in all patients placed on therapy, with a median decrease by 109 (−38 to −379) mg/g, compared with age-matched and sex-matched untreated controls that showed a 34% increase in albuminuria.

The initial voxelotor trial^70^ noted that the change in erythropoietin levels were similar across the groups, with a trend toward a lower erythropoietin level in the 1500 mg voxelotor group through week 24. This finding suggests that higher voxelotor hemoglobin occupancy, and higher hemoglobin, blunts the hypoxia-mediated induction of erythropoietin. An open-label study^75^ assessing voxelotor in participants with hepatic and renal impairment suggests no effect of kidney function on its excretion (comparable half-life values) but recommended dose reduction in severe hepatic impairment.

Like crizanlizumab, there are several ongoing trials further evaluating safety and efficacy including a randomized trial comparing risk of CKD progression in patients receiving voxelotor compared with standard of care (NCT04335721). In addition, a next generation Hb-modifying agent, GBT021601, is being studied in a phase II/III clinical trial (NCT05431088) in adults and children.

## CKD-Directed Therapeutics in SCD

### ACEi/Angiotensin Receptor Blockers

Angiotensin inhibition with ACEi and angiotensin receptor blockers, cumulatively known as renin–angiotensin–aldosterone system (RAAS) blockers, used in other albuminuric/proteinuric kidney diseases and CKD,^76–78^ have long been used in the treatment of sickle nephropathy; however, hyperkalemia has limited widespread adoption in real-world settings. Falk *et al.*,^13^ in a prospective study investigating enalapril in sickle nephropathy, found a significant decline in urinary protein excretion in ten adults after 2 weeks on enalapril. They also noted that after discontinuation of therapy, mean protein excretion remained lower than baseline. These findings were corroborated in a placebo-controlled randomized control trial of 22 adults^79^ where at 6 months of therapy with captopril, there was significant difference in the absolute (−63 mg/24 h [95% CI, 40 to 86]) and percentage (−54% [95% CI, 22% to 85%]) change in albuminuria with treatment.

More recently, angiotensin receptor blockers have been evaluated in a phase I study in which patients with SCA and persistent albuminuria (*N*=12) were placed on losartan for 1 year with renal function assessments at short (1–2 months) and long (≥12 months) time points.^80^ The albumin excretion rate decreased significantly in short-term but not at ≥12 months, although only eight patients completed long-term therapy. eGFR did not change significantly; thus, the use of losartan was believed to decrease albumin excretion with stable GFR. Another phase II multicenter trial with 6 months of losartan in adults and children with SCA met their primary end point of ≥25% reduction in albuminuria in the group of patients with microalbuminuria and macroalbuminuria.^81^

A large single-center retrospective review followed 86 patients on RAAS blockers for a median of 2.28 years compared with 68 patients not on therapy followed for 2.24 years.^82^ While not statistically significant, the odds of improved proteinuria over time were higher in the group on RAAS blockers (OR, 1.36; *P* = 0.063). There was a statistically significant difference in the rate of eGFR decline between the groups with slower rate in the group on therapy.

More long-term assessments of the efficacy and tolerance of RAAS blockade in this setting are needed, as well as prospective testing for synergism with SCD-related therapies.

### Sodium–Glucose Cotransporter-2 Inhibitors and Mineralocorticoid Receptor Antagonists

Sodium–glucose cotransporter-2 inhibitors, used as antiglycemic agents now, have indication for use in CKD and albuminuric kidney diseases. Dapagliflozin and empagliflozin have demonstrated efficacy in slowing down CKD progression, independent of diabetes diagnosis,^83,84^ and canagliflozin has shown similar efficacy in albuminuric nephropathies.^85^

Finerenone, a nonsteroidal, selective mineralocorticoid receptor antagonist, was approved for diabetic kidney disease based on the Finerenone in Reducing Kidney Failure and Disease Progression in Diabetic Kidney Disease study.^86^ The placebo-controlled randomized study of participants with CKD receiving maximum tolerated doses of RAAS blockers showed significantly decreased risk of CKD progression in the finerenone group compared with placebo group but also higher rates of hyperkalemia. Because of its proposed ability to abrogate inflammation and fibrosis, both relevant in SCD nephropathy, some have suggested finerenone may have particular benefit for this disease entity.^87^

The use of these therapies in SCD nephropathy has not yet been investigated; therefore, the benefits in this setting are unclear.

## Emerging Therapies

### Endothelin Receptor Antagonists

Endothelin (ET)-1 is a peptide that functions through binding to its receptors, ET_A_ and ET_B_, of which ET_A_ has proinflammatory, vasoconstrictive, and nociceptive actions. In SCD-associated oxidative stress, hypoxia, and vascular injury, the cellular synthesis of ET-1 is increased.^88^ ET-1 signaling contributes to development of chronic pain, vasculopathy, pulmonary hypertension, and CKD in SCD.^88^ Investigation of ET-1's role in nephropathy has shown high urinary ET-1 levels in patients with SCD and positive correlation between urinary albumin-creatinine ratio and serum ET-1 levels.^89^ In mouse models, ambrisentan, a selective ET_A_ antagonist, has demonstrated efficacy in preventing nephropathy from the level of the glomerulus to the tubules.^90^ It is now being evaluated in a phase I trial (NCT02712346) with a secondary outcome measure of urinary microalbuminuria/proteinuria. Atrasentan, another selective ET_A_ antagonist, which has been studied in diabetic nephropathy and CKD,^91^ is being evaluated (NCT04573920) for efficacy and safety in proteinuric glomerular diseases. Sparsentan, a dual angiotensin receptor and ET_A_ antagonist, has been shown to decrease proteinuria in FSGS^92^ and is undergoing additional evaluation of its long-term efficacy in FSGS (NCT03493685) and in IgA nephropathy (NCT03762850). On the basis of prespecified interim analysis of the latter study which demonstrated greater reduction in proteinuria at 36 weeks when compared with irbesartan, sparsentan recently received accelerated approval for IgA nephropathy.^93^

### Pyruvate Kinase Activators

Pyruvate kinase (PK) catalyzes the last step in glycolysis, resulting in generation of ATP. PK has a role in energy homeostasis, membrane integrity, and maintenance of 2,3-diphosphoglycerate (2,3-DPG), an intermediate of glycolysis. Elevated 2,3-DPG levels decrease oxygen affinity and pH in erythrocytes,^94,95^ which promotes deoxy-HbS polymerization and RBC sickling. PK activators increase ATP and reduce 2,3-DPG, thus reducing HbS polymerization, supporting RBC health and membrane integrity.

Mitapivat (AG-348) has demonstrated the ability to stabilize PK in sickle erythrocytes. A current phase II open-label study assessing efficacy and safety, reported on nine participants followed over 8 weeks.^96^ They saw improvements in anti-HbS polymerization, noted by a reduction in the point of sickling (a novel biomarker demonstrating when sickling is initiated *in vitro*), an increase in measured Hb-oxygen affinity, ATP/2,3-DPG ratios, and total hemoglobin, with decreases in 2,3-DPG levels and hemolysis markers. The investigators are continuing the study over an extension period as a phase II/III study (NCT05031780). Etavopivat (FT-4202) is another PK activator that is being evaluated in SCD through the Study of Etavopivat in Adults and Adolescents With Sickle Cell Disease (NCT04624659).

While these studies are not focused on nephropathy, data for GFR and albuminuria will be captured for both agents. The upstream targeting of HbS polymerization, as for voxelotor, might be predicted to result in improvements in hemolysis and anemia, with subsequent mitigation of kidney damage.

## Special Considerations in SCD Population

### Iron Chelators

Iron overload is an expected complication of chronic or recurrent transfusion therapy, indicated in many SCD-specific settings. Thus, iron chelation becomes necessary to prevent morbidity. Available chelating agents are deferoxamine, deferasirox, and deferiprone, and the choice of initiation depends on multiple factors. Deferasirox, a widely used oral chelator, has been implicated in kidney-specific adverse events ranging from dose-dependent but reversible elevations in creatinine to renal failure^97,98^; therefore, deferasirox should be used cautiously and with frequent monitoring of renal function.

### Nonsteroidal Anti-Inflammatory Drugs

Nonsteroidal anti-inflammatory drugs (NSAIDs) play a role in SCD-mediated pain management but should be used sparingly in the setting of sickle nephropathy. In the presence of nephropathy, the vasoconstrictive mechanism of NSAIDs can worsen glomerular hyperfiltration^99^ and contribute to renal insufficiency. In addition, recurrent use of NSAIDs carries the risk for acute kidney injury, which itself is a factor for developing CKD.^100^

Nephropathy, up to and including ESKD, in SCD is an important predictor of mortality. The pathophysiology of SCD-associated nephropathy is multifactorial; however, the proximate cause is hemoglobin polymerization and its downstream consequences.

**Figure 2:**
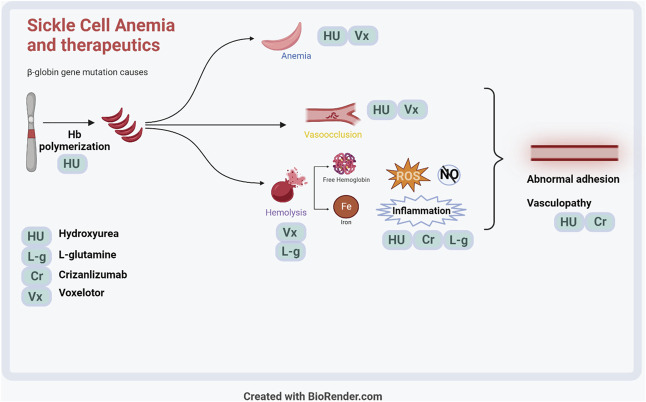
**Schematic demonstrating the major mechanisms of SCA on vasculopathy and point of effect of targeted therapeutics.** Cr, crizanlizumab; HU, hydroxyurea; L-g, l-glutamine; SCA, sickle cell anemia; Vx, voxelotor.

Newer SCD-directed therapies target aspects of the disease implicated in renal damage, including rate of RBC polymerization, anemia, hemolysis, endothelial adhesion, and vascular inflammation (Figure 2). The use of RAAS blockers, sodium–glucose cotransporter-2 inhibitors, and avoidance of nephrotoxins are universal approaches to manage CKD that are being extended to SCD-related kidney disease.

The literature contains mostly small, observational studies, so there is need for larger, longer-term, prospective, controlled, and real-world studies to better evaluate the effect of interventions on albuminuria, eGFR, tubular dysfunction, and overall renal health. Future directions for research include elucidating mechanisms of action of these therapeutics, clarifying the role of biomarkers in diagnosing renal damage and its response to therapy, and testing for potential synergism between therapeutic strategies.

## Disclosures

V.K. Derebail reports the following—consultancy: Bayer, Forma Therapeutics, Merck, Novartis, and Travere; honoraria: UpToDate; and research funding: Site PI for Clinical Trials (Boehringer Ingelheim, Chemocentryx, Gilead, Hansa, Infla-RX, Merck, Travere, and Vertex). J. Little reports the following—advisory or leadership role: Husband Tom Hostetter advisor for Tricida is not being paid for this currently or in recent past; patents or royalties: On patents currently being used at BioChip Labs and Hemex, but I receive no royalties or financial rewards from this; research funding: BlueBird Bio and GBT (Pfizer); and other interests or relationships: Member of National Association of Sickle Cell Centers and receive funding; receive funding from NHLBI; on study adjudication for Forma/Norva Nordisk Ph III Hibiscus Study. All remaining authors have nothing to disclose.
